# Simple statistical insights into the COVID-19 data of Saudi Arabia: figures prior to vaccination campaign

**DOI:** 10.12688/f1000research.52600.2

**Published:** 2021-08-04

**Authors:** Omar S. El-Masry

**Affiliations:** 1 Clinical Laboratory Sciences Department, College of Applied Medical Sciences, Imam Abdulrahman Bin Faisal university, Dammam, Rakkah, 31441, Saudi Arabia

**Keywords:** COVID-19, Case fatality rate, Death rate, outbreak

## Abstract

**Background: **COVID-19, the disease caused by the newly emerging coronavirus, SARS-COV2, is still a major health burden worldwide as it continues to spread rapidly in many countries after being contained for a while. The aim of the study was to analyze the official current disease estimates in the Kingdom of Saudi Arabia to anticipate future risks and needs.

**Methods: **Publicly available COVID-19 data published by the Saudi Ministry of Health were analyzed to extract statistical estimates of the disease. These include monthly case fatality rates, death rates/1000, comparison of death figures and regression analysis.

**Results: **The number of confirmed, recovered and deaths surged in the middle of the outbreak (June and July). The case fatality rates reported later in September-November were the highest despite the decline in the number of confirmed cases. The death rates/1000 were higher during the middle of the outbreak, where the highest numbers of deaths were recorded. The number of recovered cases was the highest as well during this time. Regression analysis showed that the number of deaths was related to that of confirmed cases, especially during the peak time. On the other hand, the number of recovered cases was related to that of confirmed cases at the beginning of the outbreak.

**Conclusion: **Statistical estimates of COVID-19 fatalities provide simple figures to understand the disease progression pattern and the health care management success in disease containment. However, the absolute numbers should never be disregarded to reflect on the real situation.

## Introduction

COVID-19, the disease caused by the newly merging coronavirus (SARS-COV2) is a major health concern worldwide currently ( Zhang et al., 2020). The pandemic is unprecedented in recent history and a lot of restrictions were imposed across the globe in an attempt to contain the spread of the infection hoping to reduce the number of carriers and, consequently, the fatality rates. This, in turn will relieve the pressure on health authorities. The outbreak has spread rapidly from its start point in the mainland of China to more than 200 countries, according to World Health Organization figures ( Zhou et al., 2020). The large number of patients who need medical care, especially those with an immediate requirement of intensive care and hospitalization resulted in overwhelming the financial, manpower and the managerial facets of healthcare centers and represented a huge burden on states ( Zhang et al., 2020).

Data available so far indicates a remarkable quick spread of the virus, suggesting a high infectivity in comparison to SARS-COV of the same genus ( Zhou et al., 2020). Regarding the virus spread, SARS-COV2, which causes COVID-19, can be transmitted via direct contact with infected surfaces or airborne droplets. Vertical transmission was not proven so far, therefore, transmission of the virus to babies from mothers is unlikely, according to the available evidence ( Zhu et al., 2020). It was also reported that SARS-COV2 has a higher morbidity and mortality, and higher fatality rates than SARS-COV. At this end, COVID-19 patients suffer from severe suppression of cellular-mediated immune response to the virus as evident by the low count of CD3 ^+^, CD4 ^+^, and CD8 ^+^ cells ( Chen et al., 2020). In this respect, SARS-COV, downregulates interferon (IFN) regulatory factor 3 (IRF-3), which ameliorates the antiviral activity mediated by IFN α and β axis; this scenario might also contribute to the suppression of antiviral activity in COVID-19 patients ( Kuri and Weber, 2010). In a similar respect, early reports this year indicated that SARS-COV2 is detectable in clinical samples of non-survivors throughout the clinical course of the disease ( Weiss and Murdoch, 2020). Moreover, an American study postulated that ethnicity and gender differences might have a potential influence on hospitalization and mortality of COVID-19 patients ( Price-Haywood et al., 2020).

The aim of the current study was to provide up to date simple statistical estimates and insights into the official numbers published by the Saudi Ministry of Health and establish an overview to understand the epidemiological data of COVID-19 in the Kingdom of Saudi Arabia. This might help to set future action plans to improve the overall outcomes of disease management.

## Methods

This is a data-based study in which the official data on COVID-19 disease that were published by the Saudi Ministry of Health on COVID-19 dashboard were analyzed. Data are available from: https://covid19.moh.gov.sa/. The data published on the dashboard are presented as graphs showing the numbers of confirmed cases, recovered cases and deaths day by day. The numbers are updated on a daily basis.

The following data were extracted from the dashboard for this study: minimum and maximum numbers for confirmed cases of COVID-19, recovered cases of COVID-19, and deaths relating to COVID-19 reported for March-November 2020 The total number of people living in Saudi Arabia was determined according to the estimates of the United Nations that were published on the Worldometer website: https://www.worldometers.info/.

The total number of deaths in each month and case fatality rates were compared between the months in 2020, and death rates/1000 of the population were calculated. Regression analysis was also performed to underline the relationship between different figures reported for each month including the relationships between: (1) the number of confirmed cases (daily reported infections in each month) and the number of the recovered cases (daily reported recoveries in each month), and (2) the number of confirmed cases and the number of monthly reported COVID-19-related deaths (daily reported deaths in each month).

Statistical presentations and calculations were performed using Graphpad Prism (version 7). The data were tested for normality and the Kruskal Wallis test was employed for comparison between groups, followed by Dunn’s multiple comparison test. The difference was considered statistically significant at p ≤ 0.05.

## Results

Table 1 shows the minimum and maximum numbers recorded for COVID-19 confirmed cases, recovered cases and deaths for each month of 2020. The maximum numbers of confirmed cases were recorded during June, while the maximum recovered cases and deaths were recorded in July. There was an increase in the number of confirmed cases from March-June, and then the numbers started to decrease during July-November. The low number recorded in the first one or two months might be associated with the total number of performed tests, where the capacity of health authorities worldwide may not have been fully prepared for such a surprising pandemic.

**Table 1.** Numbers of confirmed and recovered cases, and deaths related to COVID-19 for 2020 (March-November) in the Kingdom of Saudi Arabia.

	Month								
	March	April	May	June	July	August	September	October	November
**Confirmed cases**									
Minimum	0	154	1362	1869	1573	898	403	323	217
Maximum	205	1351	2840	4919	4207	1569	833	501	473
Sum	1563	22543	63045	107083	83253	39192	18337	12559	9967
**Recovered cases**									
Minimum	0	19	210	806	1742	718	592	335	357
Maximum	50	392	3559	9651	7718	5426	1454	626	495
Sum	165	3390	60751	76457	104788	54966	28232	14490	12940
**Deaths**									
Minimum	0	3	7	24	20	27	24	14	11
Maximum	4	9	23	50	58	42	37	29	20
Sum	10	159	356	1173	1189	1042	865	626	487

### Comparing death figures

Table 2 shows the statistical comparison between the numbers of daily deaths recorded in each month (March-November). As the data were not following the Gaussian distribution, the Kruskal Wallis test was performed, followed by Dunn’s multiple comparison test, which indicated that the numbers of deaths recorded in May-November were significantly higher than the numbers recorded in March (p < 0.0001; except for May, p < 0.05). The difference between the numbers of deaths recorded in April and May was not significant, whilst the numbers recorded in June-October were significantly higher than April and May figures (p < 0.0001), except for the comparison between May and October, the difference was not significant, perhaps due to the reduction in number of deaths in October and November. Also, the numbers of deaths in October and November were significantly lower than that reported in June-August indicating the gradual reduction in the total number of deaths. In addition, there was no significant difference between, June, July, August and September figures, which might explain that the average reduction in the death numbers in September was not the desirable one as compared to the reduction observed in October and November. In other words, despite the decline in the number of confirmed cases in September in comparison to June-August period, the decline in the reported deaths in this month was not significantly different from death figures in June-August.

**Table 2.** Summary of the Kruskal Wallis statistics comparing the number of deaths in each month (Dunn’s multiple comparison test).

	Month									
**Month**		March	April	May	June	July	August	September	October	November
	March		NS	*	****	****	****	****	****	***
	April	NS		NS	****	****	****	****	**	NS
	May	*	NS		****	****	****	****	NS	NS
	June	****	****	****		NS	NS	NS	****	****
	July	****	****	****	NS		NS	NS	***	****
	August	****	****	****	NS	NS		NS	**	****
	September	****	****	****	NS	NS	NS		NS	*
	October	****	**	NS	****	***	**	NS		NS
	November	***	NS	NS	****	****	****	*	NS	

NS: Not significant (p value > 0.05), *: Significant difference (p value < 0.05), **: Significant difference (p value < 0.01), ***: Significant difference (p value < 0.001), ****: Significant difference (p value < 0.0001)

### Case fatality rates and death rates/1000

The case fatality rate is defined as the total number of deaths from a particular cause (COVID-19, in this case)/total number of cases x 100. The case fatality rates in each month are presented in Table 3. The lowest numbers of confirmed cases were reported throughout March and case fatality rate was higher than the rate reported for May (0.64 vs. 0.56%), perhaps due to the surprising outbreak of the pandemic and lack of the necessary preparations to confront such situations. Likewise, the rate in April was higher than that of May (0.7). Also, despite the declining figures of confirmed disease and death cases until November following the surge in June and July, the case fatality rates in September-November were the highest (4.71%, 4.98%, and 4.88%, respectively), higher than June and July, wherein the highest numbers of confirmed cases and deaths were reported. This might suggest the poor prognosis in the patient group of non-survivors, or the increased virulence of the virus, but we have to bear in mind the large number of cases in June and July in comparison to the following months. In the same context, the death rates/1000 of the population ( Table 3) were also calculated. The results suggested that the highest death rates/1000 were recorded in June, July and August (0.03). Obviously, the lowest figures of this parameter were seen in March and April. In addition, the figures of September-November were less than that of June and July, despite the highest case fatality rates observed in these months.

**Table 3.** Case fatality rates and death rate/1000 related to COVID-19 for 2020 (March-November) in the Kingdom of Saudi Arabia.

Month	Total number of deaths	Case fatality rate (%)	Death rate/1000
March	10	0.64	0.00028
April	159	0.7	0.0045
May	356	0.56	0.0102
June	1173	1.09	**0.0337**
July	1189	1.43	**0.034**
August	1042	2.66	0.03
September	865	**4.71**	0.025
October	626	**4.98**	0.018
November	487	**4.88**	0.014

*Case fatality rate = Total number of deaths from Covid-19/total number of confirmed cases*100. Death rate/1000 = Total number of recorded deaths from Covid-19/the number of Saudi population*1000.

* The Saudi population number was estimated at 34813871 at mid-year according to the UN estimates.

### Relationship between the number of confirmed cases and death figures

**Figure 1.**
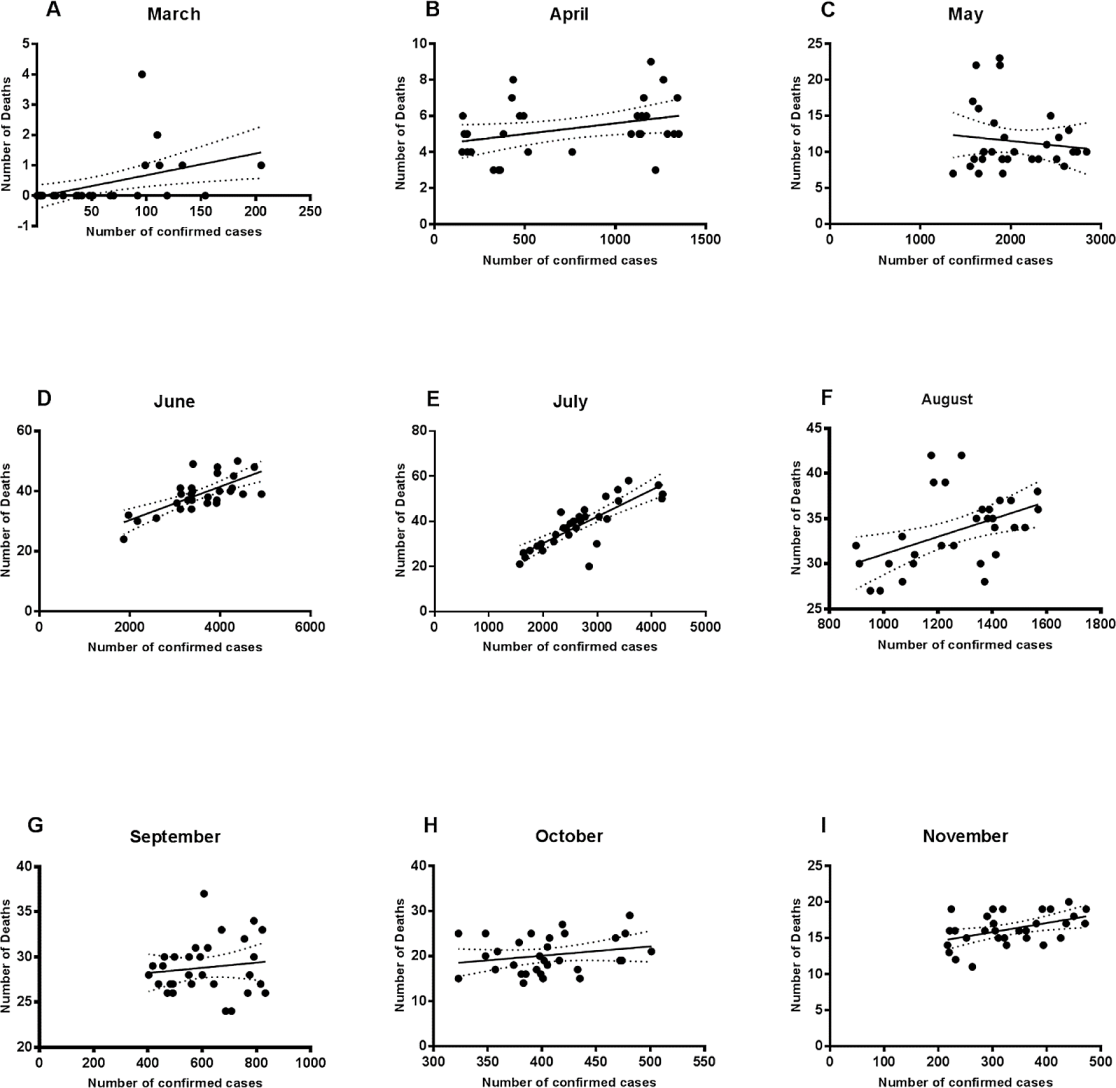
Regression analysis of the number of confirmed cases and deaths. Regression analysis showed that the number of deaths could be weakly related to the number of confirmed cases in March (A), August (F), and November (I). The relationship was significantly strong in June (D) and July (E).

The relationship between the number of confirmed cases and the number of deaths in each month was investigated by regression analysis ( Figure 1). Using this type of analysis we can understand how the change in the number of confirmed cases of COVID-19 is related to the number of deaths. The analysis showed that the slope was significantly non-zero (this means that the relationship between the tested variables was represented as a line with an apparent steepness, where the number of deaths (y) changes according to the change in the number of confirmed cases (x); simply the line is not horizontal) for March, though the association relationship was weak (R = 0.218; p value = 0.009). The slope was close to zero for April, May, and September (R = 0.12, 0.01, and 0.01; p value = 0.06, 0.51, and 0.49), which means that the change in the number of confirmed cases in these months was not associated with the change in the number of deaths. Regarding the figures of regression analysis in June and July, the best fit graph showed a stronger significant association between the number of confirmed cases and deaths (R = 0.5, and 0.68; p value = < 0.0001) as compared to the figures in the other months. August results showed a weaker positive association and a non-zero slope of the best fit line (R = 0.21 and p value = 0.008). The slope was close to zero for October indicating a lack of the relationship, while there was a weak association for November figures (R = 0.206 and p value = 0.012).

### Relationship between the number of confirmed cases and recovery figures

**Figure 2.**
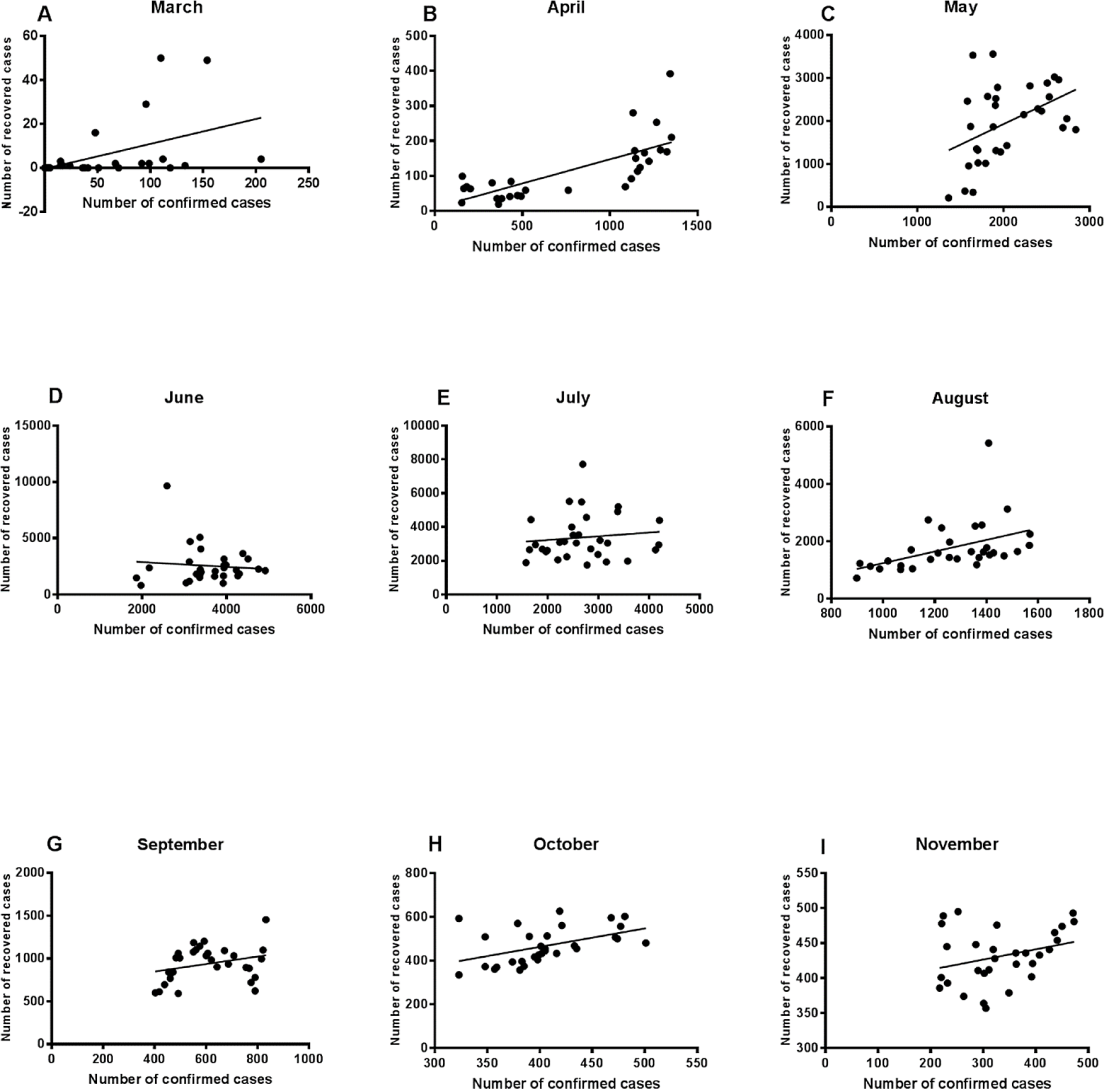
Regression analysis of the number of confirmed cases and recovered cases. Regression analysis showed that the number of recovered cases could be significantly related to the number of confirmed cases in March (A), April (B) and May (C). A weaker association was reported for August (F) and October (H).

Figure 2 shows the regression analysis results in March-November for the assessment of the relationship between the number of the confirmed cases and recovered cases. The results showed that the number of the recovered cases of COVID-19 patients in March, April, May and August could be explained by the number of confirmed cases (R = 0.22, 0.54, 0.2, and 0.2; p values < 0.008, 0.0001, 0.01, and 0.01). The strongest relationship between the number of confirmed cases and the number of the recovered cases was then observed during April, where 50% of the recovery figures could be explained by the confirmed cases figures. The slope was close to zero for June, July and September analysis, which means that the number of the recovered cases are not related to the numbers of the confirmed cases, although June and July witnessed the highest number of the recovered cases (R = 0.009, 0.014, and 0.08; p value = 0.52 and 0.12). However, these results are consistent with the high death figures observed in June, July and September. There was a significant relationship between the numbers of the recovered and the confirmed cases in October (R = 0.224 and p value = 0.007). The figures for November showed that the relationship was not significant.

## Discussion

As the recently emerging pandemic of COVID-19 continues to attract attention all over the world with uncertainty about when the disease will vanish and cease new waves, the need continues to grow to assess the capacity of health care authorities to cope with the current challenge and be ready for all possibilities. In this respect, the assessment of the past and present situation data is important for accurate need assessment for future preparation. Therefore, the main aim of the current study was to analyze the data to conclude some statistical figures that could help to understand how effective the management of the situation was, and what should we do for better clinical management outcomes of the COVID-19 crisis. The results showed that the numbers of confirmed deaths reached the peak during June and July, with the highest death rates/1000 of the population. Also, despite the decrease in the number of the confirmed deaths in September-November, the case fatality rates were the highest throughout the period from March-November, which also showed a small number of confirmed cases that might have resulted in the high case fatality rate. The world is still overwhelmed with the pandemic; however, the situation seems to be under control in Saudi Arabia, despite the high case fatality rates recorded in September-November. Case fatality could be affected with multiple factors, such as patients’ age, comorbidities or virus virulence and immune response efficacy in patients. To this end, other data, such as patients’ demographics, comorbidities, medical history, autopsy reports and detailed lab records and clinical follow up data should be available for a more comprehensive figure. In this regard, it was reported that up to 81% of COVID-19 patients might suffer only from a mild disease, which does not require hospitalization ( Price-Haywood et al., 2020). Having said that, the other 19%, which is a proportion large enough to overwhelm the health care sector, might suffer from severe symptoms that impose hospitalization and here is where mortality rates could increase as a result of poor clinical management.

It was also recently reported that the average case fatality rate of COVID-19 is less than 5% ( Sun et al., 2020). This estimate suggests that the case fatality rates reported in the current study are within the global figures. The surprising hit of the pandemic and/or the virulence of the virus strain and its quick spread needed some time for the governments to confine the situation. In this regard, many comorbidities were linked to the increased severity and mortality of COVID-19 patients, including diabetes mellitus ( Kumar et al., 2020). It is of importance to denote that diabetes mellitus is prevalent in Saudi Arabia ( Alotaibi et al., 2017). However, more data should be available on COVID-19 casualties before assuming any link between the disease and COVID-19 mortality figures presented herein. It might be also of importance to mention that the case fatality rates of COVID-19 are lower than that of Severe Acute Respiratory Syndrome (SARS) and Middle East Respiratory Syndrome (MERS) ( Sun et al., 2020), but we have to bear in mind that the number of cases of COVID-19 patients is much higher than that recorded for the other two counterparts. Similar case fatality rates were reported for both China and Italy (~2.3) at the beginning of the pandemic, with comorbidities that might have contributed significantly to mortality in elderly patients ( Porcheddu et al., 2020). These figures are almost similar to the average case fatalities reported in the period (March-September) in Saudi Arabia. In line with the current study, Boretti compared some figures of the outbreak between Saudi Arabia and the United Kingdom in the middle of the outbreak; Boretti reported that the number of cases per million, the number of deaths per million and the number of newly daily deaths per million were much less in Saudi Arabia than those in the United Kingdom ( Boretti, 2020).

In order to give a brief account on the figures post vaccination campaign, the case fatality rates were calculated from the officially published figures in the first 6 months in 2021 (January-June). The number of newly recorded cases escalated from ~5000 cases in January to ~37000 in June. This means that following flattening of the curve at the end of 2020, there was an ascending increase in the number of cases again after the start of the vaccination campaign. However, the number of cases still much less than the numbers recorded in the same months in 2020 before vaccination. The case fatality rate in January 2021 was 0.03 (3%), whilst that calculated for February-June was 0.01 (1%), which is similar to that recorded for June 2020; however, there was a clear reduction in the number of confirmed cases and deaths in 2021. Whether the reduction in confirmed cases and deaths numbers is due to vaccination or other factors, this is not confirmed due to lack of information about the number of vaccinated people who contracted the infection nor those who died after being vaccinated. Therefore, we have to wait the release of all official figures and information before we make a final conclusion about the efficacy of vaccination.

In conclusion, the results presented in the current study suggested that the fatality rates of COVID-19 mortality and their association with the number of confirmed cases and the recovery figures should be always seen in the same frame with the absolute figures to reflect the real situation to the policy makers for better needs assessment, future planning and prompt response in crisis time. Amongst the limitations of the current study, and maybe other related published articles, are the lack of information on cases and deaths age groups, comorbidities, ethnicity, and medical history that might have affected disease progression and mortality rates. The availability of such information could help to clarify risk factors and those predisposed for a severe disease clinical course. Also, we have to bear in mind that the number of COVID-19 cases are much higher than that of the disease counterparts SARS and MERS when we look at the fatality rates.

## Data availability

All data presented herein were extracted by analyzing the publicly available data on COVID-19 that were uploaded to the COVID-19 data portal established by the Saudi Ministry of Health: https://covid19.moh.gov.sa/.
