# Supplementary material for: Simple statistical insights into the COVID-19 data of Saudi Arabia: figures prior to vaccination campaign
Source: F1000Res. 2021 Aug 4;10:410. Originally published 2021 May 21. [Version 2] doi: 10.12688/f1000research.52600.2 (PMC11358676; doi:10.12688/f1000research.52600.2)
Supplement: Peer review history

# Reviewer report: Reviewer response for version 2

**DOI:** 10.5256/f1000research.59066.r234875

**Published:** 2024-08-28

**Authors:** Mohammad Nayeem Hasan

**Affiliations:** 1 Department of Statistics, Shahjalal University of Science & Technology, Sylhet, Bangladesh

The study entitled “Simple statistical insights into the COVID-19 data of Saudi Arabia: figures prior to vaccination campaign” tries to evaluate the the official current disease estimates in the Kingdom of Saudi Arabia to anticipate future risks and needs. The manuscript is well organized and the methodology is considerable for acceptance. However, I suggest some improvements below:

 1.Provide some research gaps in the introduction.

 2.Please explain all variables and how you categorized them. If needed, provide references.

 3.Please follow some recently published papers related to your study from high-impact journals.

 4.Please explain, how you reached the final model, do you use any model evaluation techniques like AUROC, multicollinearity, feature selection, P-value, etc.?

 5.Please, explain the discussion based on your findings in brief. You can follow them: [Ref 1,2,3 and 4]

Is the work clearly and accurately presented and does it cite the current literature?

Yes

If applicable, is the statistical analysis and its interpretation appropriate?

Yes

Are all the source data underlying the results available to ensure full reproducibility?

Yes

Is the study design appropriate and is the work technically sound?

Yes

Are the conclusions drawn adequately supported by the results?

Yes

Are sufficient details of methods and analysis provided to allow replication by others?

Yes

Reviewer Expertise:

Public Health

I confirm that I have read this submission and believe that I have an appropriate level of expertise to confirm that it is of an acceptable scientific standard, however I have significant reservations, as outlined above.

## References

[1] : Effects of fine particulate matter (PM2.5) and meteorological factors on the daily confirmed cases of COVID-19 in Bangkok during 2020–2021, Thailand. *Case Studies in Chemical and Environmental Engineering* .2023;8: 10.1016/j.cscee.2023.100410 10.1016/j.cscee.2023.100410 PMC1028657338620170

[2] : The disproportionate case-fatality ratio of COVID-19 between countries with the highest vaccination rates and the rest of the world. *IJID Reg* .2023;6: 10.1016/j.ijregi.2023.01.011 159-166 10.1016/j.ijregi.2023.01.011 36721772 PMC9881127

[3] : Two Decades of Endemic Dengue in Bangladesh (2000-2022): Trends, Seasonality, and impact of Temperature and Rainfall Patterns on transmission dynamics. *medRxiv* .2023; 10.1101/2023.07.16.23292380 10.1101/2023.07.16.23292380 PMC1093616738253990

[4] : The disproportionate case-fatality ratio of COVID-19 between countries with the highest vaccination rates and the rest of the world. *IJID Reg* .2023;6: 10.1016/j.ijregi.2023.01.011 159-166 10.1016/j.ijregi.2023.01.011 36721772 PMC9881127

# Reviewer report: Reviewer response for version 2

**DOI:** 10.5256/f1000research.59066.r234882

**Published:** 2024-02-14

**Authors:** Ahmad Abubakar Suleiman

**Affiliations:** 1 Department of Statistics, Kano University of Science and Technology, Wudil, Kano, Nigeria; 2 Fundamental and Applied Science, Universiti Teknologi PETRONAS, Perak, Malaysia

The problem is seen as new and will make a considerable impact in the scientific community, and the paper’s subject could be interesting for readers of journal. However, some major concerns should be addressed before the final acceptance of the article.

1. The main contributions by authors are insufficient, unclear, and What are the new findings and new contributions?  In the introduction, no context or motivation are provided, and the novelty of the present work is not discussed.
2. The paper should be carefully revised for punctuation, grammar, and spelling mistakes.
3. The paper doesn't bring much above what is already known, although it could have some value if the authors provided clinical and nonpharmaceutical measures taken by the Kingdom of Saudi Arabia prior to the vaccination campaign.
4. The authors have clearly mentioned the source of the datasets used in this investigation. Please add the access dates (format: Date Month Year), e.g., accessed on 1 January 2020.
5. Mathematical equations for regression analysis must be provided:

1. The number of confirmed cases (x) and the number of recovered cases (y)
2. The number of confirmed cases (x) and the number of monthly reported COVID-19-related deaths (y). Add this reference (https://www.sciencedirect.com/science/article/pii/S2590053620301087)

The same thing must be done to obtain Kruskal-Wallis and Dunn’s tests. Please write the case fatality rate formula correctly as an equation and provide an equation number in all cases. Put all equations in the Methods section.

1. Simple models seem to have predicted the trend well in the near future; however, the data changed with the date. So, the regression model adjusted for the date should fit the data more robustly in the real situation. Suggesting the conclusion and recommendations using polynomial model-based results were drawn.
2. You may cite this reference to support your Results Section (https://www.mdpi.com/2071-1050/15/18/13647).
3. The conclusion section must be more precise. Be specific while writing your main findings.
4. References must be updated.

Is the work clearly and accurately presented and does it cite the current literature?

Partly

If applicable, is the statistical analysis and its interpretation appropriate?

Partly

Are all the source data underlying the results available to ensure full reproducibility?

Yes

Is the study design appropriate and is the work technically sound?

Yes

Are the conclusions drawn adequately supported by the results?

Yes

Are sufficient details of methods and analysis provided to allow replication by others?

Partly

Reviewer Expertise:

Statistics

I confirm that I have read this submission and believe that I have an appropriate level of expertise to confirm that it is of an acceptable scientific standard, however I have significant reservations, as outlined above.

## References

[1] : Estimation of the case fatality rate of COVID-19 epidemiological data in Nigeria using statistical regression analysis. *Biosaf Health* .2021;3(1) : 10.1016/j.bsheal.2020.09.003 4-7 10.1016/j.bsheal.2020.09.003 32935084 PMC7482605

[2] : Tropically Adapted Passive Building: A Descriptive-Analytical Approach Using Multiple Linear Regression and Probability Models to Predict Indoor Temperature. *Sustainability* .2023;15(18) : 10.3390/su151813647 10.3390/su151813647

[3] : Forecasting the Volatility of Real Residential Property Prices in Malaysia: A Comparison of Garch Models. *Real Estate Management and Valuation* .2023;31(3) : 10.2478/remav-2023-0018 20-31 10.2478/remav-2023-0018

# Reviewer report: Reviewer response for version 2

**DOI:** 10.5256/f1000research.59066.r91211

**Published:** 2021-08-04

**Authors:** Yahia Ali Kaabi

**Affiliations:** 1 Department of Medical Laboratory Technology, Faculty of Applied Medical Sciences, Jazan University, Jazan, Saudi Arabia

The author provided all needed amendments successfully.

Is the work clearly and accurately presented and does it cite the current literature?

Yes

If applicable, is the statistical analysis and its interpretation appropriate?

Yes

Are all the source data underlying the results available to ensure full reproducibility?

Yes

Is the study design appropriate and is the work technically sound?

Yes

Are the conclusions drawn adequately supported by the results?

Partly

Are sufficient details of methods and analysis provided to allow replication by others?

Yes

Reviewer Expertise:

NA

I confirm that I have read this submission and believe that I have an appropriate level of expertise to confirm that it is of an acceptable scientific standard.

# Reviewer report: Reviewer response for version 1

**DOI:** 10.5256/f1000research.55900.r88338

**Published:** 2021-07-15

**Authors:** Yahia Ali Kaabi

**Affiliations:** 1 Department of Medical Laboratory Technology, Faculty of Applied Medical Sciences, Jazan University, Jazan, Saudi Arabia

The manuscript titled “Simple statistical insights into the COVID-19 data of Saudi Arabia: figures prior to vaccination campaign” is a good attempt to brief the disease figures in Saudi Arabia by providing a statistical summary of death and recovery rates, as well as the number of confirmed cases throughout the past year before starting vaccination.

 The article rationale is good and the author summarized the official figures of COVID-19 in Saudi Arabia that were published by the Ministry of Health in a simple way for the readership. This simple presentation of COVID-19 data is needed to understand the graph of the outbreak and what health authorities did to contain the outbreak. Availability of such figures is also needed to help health authorities anticipate their future needs and put plans for future actions and precautionary measures.

 The introduction provided a sufficient background on Coronavirus and the study aim was clearly mentioned at the end of this section. However, I would suggest adding one paragraph to provide an account on the current situation worldwide and in Saudi Arabia.

 The methods section was concise, but clear and data source and all statistical analysis were stated and justified by the author.

 The results were also clearly presented; in particular the detailed comparison of death figures in each month was important when correlating to the number of new incidents/monthly. In addition, comparison between local figures and international figures highlighted the similarity between Saudi Arabia and many other international figures, which was important to show how far or close are we from international statistics. It would be good if the author could also explain how death rate/1000 is calculated below table 3, as done for case fatality rate. In Figure 2, the legend should be corrected to describe the recovered cases not deaths.

 I would also suggest adding one paragraph to the discussion section to report on the figures after vaccination campaign till now, just to provide a simple comparison between the situations before and after vaccination to assess the success of vaccination in containing the mortality effects of COVID-19 in the country.

Is the work clearly and accurately presented and does it cite the current literature?

Yes

If applicable, is the statistical analysis and its interpretation appropriate?

Yes

Are all the source data underlying the results available to ensure full reproducibility?

Yes

Is the study design appropriate and is the work technically sound?

Yes

Are the conclusions drawn adequately supported by the results?

Partly

Are sufficient details of methods and analysis provided to allow replication by others?

Yes

Reviewer Expertise:

NA

I confirm that I have read this submission and believe that I have an appropriate level of expertise to confirm that it is of an acceptable scientific standard, however I have significant reservations, as outlined above.

# Response

**Published:** 2021-07-27

**Authors:** Omar S. El-Masry

**Affiliations:** Imam Abdulrahman Bin Faisal University, Saudi Arabia

In response to the valuable comment on the introduction section, I think adding more information on the current situation in the introduction could also require a change in the paper’s title, which mainly focuses on the figures prior to the vaccination campaign in the Kingdom. However, the current figures will be briefly discussed in the discussion section.

 Thank you for your valuable comment on the results section, the equation for death rate/1000 was added below table 3 and Figure 2 legend was corrected as indicated, thank you.

 The following paragraph was added to the discussion section as recommended; I focused on the case fatality rate, which reflects directly on the number of confirmed cases and the number of deaths, death rate /1000 was not calculated as the number of Saudi population in 2021 is different than that in 2020 therefore comparison is not significant.

 In order to give a brief account on the figures post-vaccination campaign, the case fatality rates were calculated from the officially published figures in the first 6 months in 2021 (January-June). The number of newly recorded cases escalated from ~5000 cases in January to ~37000 in June. This means that following flattening of the curve at the end of 2020, there was an ascending increase in the number of cases again after the start of the vaccination campaign. However, the number of cases is still much less than the numbers recorded in the same months in 2020 before vaccination.  The case fatality rate in January 2021 was 0.03 (3%), whilst that calculated for February-June was 0.01 (1%), which is similar to that recorded for June 2020; however, there was a clear reduction in the number of confirmed cases and deaths in 2021. Whether the reduction in confirmed cases and deaths numbers is due to vaccination or other factors, this is not confirmed due to lack of information about the number of vaccinated people who contracted the infection nor those who died after being vaccinated. Therefore, we have to wait for the release of all official figures and information before we make a final conclusion about the efficacy of vaccination. 

 An updated version was submitted for your kind review.
